# 6,7-di-O-acetylsinococuline (FK-3000) induces G_2_/M phase arrest in breast carcinomas through p38 MAPK phosphorylation and CDC25B dephosphorylation

**DOI:** 10.3892/ijo.2014.2739

**Published:** 2014-11-07

**Authors:** YONG-CHUN LI, BONG-HEE KIM, SOON-CHANG CHO, MI-AE BANG, SUNMIN KIM, DAE-HUN PARK

**Affiliations:** 1 Department of Oriental Medicine Materials, Dongshin University, Naju, Jeonnam 520-714, Republic of Korea; 2 School of Pharmaceutical Science, Zhengzhou University, Zhengzhou, Henan 450001, P.R. China; 3 Chungnam National University, Yuseong, Daejeon 305-764, Republic of Korea; 4 Research Institute, NaturePureKorea Inc., Damyang 517-803, Republic of Korea; 5 Food Industry Development Team, Jeonnam Biofood Technology Center, Naju, Republic of Korea

**Keywords:** 6,7-di-O-acetylsinococuline (FK-3000), human carcinoma cell lines, cell cycle arrest, p38 MAPK phosphorylation, CDC25B dephosphorylation, apoptosis

## Abstract

We evaluated the cytostatic effect of 6,7-di-O-acetylsinococuline (FK-3000) isolated from *Stephania delavayi* Diels. against breast carcinoma cell lines MDA-MB-231 and MCF-7. FK-3000 suppressed CDC25B phosphorylation directly and indirectly via p38 MAPK phosphorylation. CDC25B dephosphorylation decreased levels of cyclin B and phospho-CDC-2, and ultimately induced cell cycle arrest at the G_2_/M phase. The p38 MAPK inhibitor, SB 239063 blocked FK-3000-induced p38 MAPK phosphorylation and nuclear accumulation, but did not completely rescue cell death. Conclusively FK-3000 exerts its antiproliferative effect through two pathways: i) G_2_/M cell cycle arrest via downregulation of cyclin B and phospho-CDC2 by p38 MAPK phosphorylation and CDC25B dephosphorylation, and ii) p38 MAPK-independent induction of apoptosis.

## Introduction

In 2014, the American Cancer Society reported that cancer was the second leading cause of death in the USA, estimating that >1,665,540 new cancer cases would be diagnosed that year and 585,720 cancer deaths would occur in the USA. To develop effective treatments, scientists have isolated anticancer agents from natural materials and identified a number of promising drug candidates (1).

In the search for potential therapies, we screened 509 natural products and found 14 compounds that demonstrate anticancer properties. One of these natural products was 6,7-di-O-acetylsinococuline (FK-3000), which was isolated from *Stephania delavayi* Diels. A literature search of the pharmaceutical properties of FK-3000 revealed a compound isolated from *S. cepharantha* that inhibits nuclear factor κB activity (2) and exhibits antiviral effects against herpes simplex virus type-1 (3), by inhibiting DNA synthesis (4), and against human immunodeficiency virus type-1 (5).

Mammalian cell division is controlled by cyclins and cyclin-dependent kinases (CDKs), which form various cyclin-CDK heterodimeric complexes (protein kinase holoenzymes) that regulate different phases of the cell cycle. Positive regulators of CDK function are upregulated in most cancer cells, whereas the expression of negative regulators are downregulated. Accordingly, cyclin D1, CDK4, cyclin E, cyclin A, and Wee1 are upregulated in the Long-Evans Cinnamon rat model of hepatocellular carcinoma (6); CDK4 plays a pivotal role in the progression from preneoplastic to neoplastic status in diethylnitrosamine-induced hepatocellular carcinoma in rats (7); increased expression of cell cycle regulatory proteins and kinase activities of cyclin D1, CDK4, cyclin E, cyclin A, and Wee1 was revealed by epidemiological studies of patients with liver disease (8); and inhibitors of cell division cycle 25 (CDC25) phosphatases have shown promise as anticancer agents (9). Targeting CDKs or cell cycle protein kinases is an important strategy in the discovery of novel anticancer drugs, and several preclinical and clinical trials are assessing these proteins as targets (10).

In humans, there are three homologues of CDC25: CDC25A, CDC25B, and CDC265C. In an earlier CDC25 regulation model, CDC25A controls the G_1_/S cell cycle transition, and CDC25B and CDC25C control mitosis (11) but in recent studies it was found that all three homologues have function to control both G_1_/S and G_2_/M phase transitions and mitosis (12). CDC25B facilitates dephosphorylation of the key cell cycle regulator CDC2 (also called CDK1) at Tyr15 or Thr14, thereby initiating the G_2_/M transition (13). Moreover, CDC25B is overexpressed in most tumor types, including head and neck, ovary, colon, and breast cancers, suggesting its potential as a target for novel anticancer drugs (14). Examples of CDC25 inhibitors include the compound BN82002, which strongly inhibits CDC25 activation and delays cell cycle progression at the G_1_/S transition, in S phase, and at the G_2_/M transition (15); silymarin and silibinin, which arrest human prostate cancer PC3 cells at the G_1_ and G_2_/M phases and specifically decrease levels of cyclin B1, cyclin A, phospho-CDC2 (Tyr15), and CDC2 (16); naphthofurandione 3-benzoyl-naphtho[1,2-*b*] furan-4,5-dione, which inhibits recombinant CDC25B *in vitro*, exhibits 96-h half maximal inhibitory concentration (IC_50_) of 6.5 μM against MCF-7 cells and 1.2 μM against MDA-MB-231 cells, and causes G_1_/S and G_2_/M phase arrest (17); BN82685, which inhibits recombinant CDC25A, -B, and -C, and inhibits growth of the human pancreatic tumor Mia PaCa-2 xenografted in athymic mice (18); and IRC-083864, which inhibits cell proliferation by p21 induction and apoptosis (19).

Activation of p38 mitogen-activated protein kinase (p38 MAPK) arrests cells in the G_2_/M phase by inhibiting CDC25B phosphorylation (19) and blocking participation of the CDC2/cyclin B complex in the G_2_/M phase transition (20,21).

In the present study, we investigated the antiproliferative mechanisms of FK-3000 by examining its effect on cell cycle regulatory proteins. Our results show that FK-3000 decreased levels of phosphorylated CDC25B (phospho-CDC25B) but neither CDC25A nor CDC25C, and induced G_2_/M phase arrest in human breast carcinoma cell lines MDA-MB231 and MCF-7.

## Materials and methods

### Isolation of 6,7,-di-O-acetylsinococuline (FK-3000)

The methanol extract (1 g) of *S. delavayi* Diels. was separated by chromatography on a Sephadex LH-20 column (GE Healthcare, Uppsala, Sweden, 40i.d.x860 mm, 25–100 μm, eluted with methanol). Fraction 3 (700 mg) was further purified by C18 high performance liquid chromatography [YMC-Pack Pro, YMC GmbH, Leicestershire, UK, S-5 μm, 20i.d.x250 mm) with 10–30% aqueous acetonitrile (0.05% trifluoroacetic acid, Sigma-Aldrich Co., St. Louis, MO, USA) for 90 min at 7 ml/min, yielding FK-3000 (76 mg; retention time, 82.14 min) as pale brown needles. The ^1^H, ^13^C, and two-dimensional nuclear magnetic resonance (2D NMR) spectra of the isolate were in good agreement with those of FK-3000 isolated from *S. cepharantha* (22).

### Cell culture and cell viability assay

The human breast carcinoma cell lines MDA-MB-231 and MCF-7 were obtained from the Korean Cell Line Bank (Seoul, Korea). Cells were cultivated in RPMI-1640 (Gibco/BRL, Grand Island, NY, USA) containing 10% fetal bovine serum (Gibco/BRL), 2 mg/ml sodium bicarbonate (Gibco/BRL), 100 U/ml penicillin (Gibco/BRL), and 100 μg/ml streptomycin (Gibco/BRL).

Cells were seeded in 96-well plates (1.5×10^4^ cells/well) and incubated at 37°C in a 5% CO_2_ atmosphere. To determine the IC_50_ of FK-3000, MDA-MB-231 and MCF-7 cells were treated with 0.1% dimethyl sulfoxide (DMSO; vehicle only control) (Sigma-Aldrich Co.) or FK-3000 (0–5 μg/ml) 24 h after seeding. Cell proliferation was analyzed after 24 and 48 h using the cell counting kit-8 (Dojindo Molecular Technologies, Rockville, MD, USA) according to the manufacturer’s instructions.

To evaluate cell viability, cells were treated with 0.1% DMSO, the 48 h IC_50_ of FK-3000 (0.52 μg/ml for MDA-MB-231 cells; 0.77 μg/ml for MCF-7 cells), 5.0 μM *trans*-1-(4-hydroxy-cyclohexyl)-4-(4-fluorophenyl)-5-(2-methoxypyridimidin-4-yl)-imidazole (p38 MAPK inhibitor SB 239063, Sigma-Aldrich Co.), or cotreated with the 48 h IC_50_ of FK-3000 and 5.0 μM SB 239063. Cell viability was evaluated after 48 h using the cell counting kit-8 (Dojindo Molecular Technologies). All experiments were performed in quadruplicate on different days.

### Cell cycle distribution assay

Cells were seeded in 100-mm culture dishes (1.0×10^6^ cells/well). After attachment, cells were synchronized by fetal bovine serum withdrawal for 6 h and then treated in quadruplicate with DMSO only, FK-3000 (MDA-MB-231 cells, 0.5 μg/ml; MCF-7 cells, 0.7 μg/ml), SB 239063 (both cell lines, 5.0 μM), or combination treatment (MDA-MB-231 cells, 0.5 μg/ml FK-3000 + 5.0 μM SB 239063; MCF-7 cells, 0.7 μg/ml FK-3000 + 5.0 μM SB 239063). Cells were harvested after 24 or 48 h of treatment and fixed with ice-cold 70% ethanol at 4°C. After 24 h, the fixed cells were centrifuged at 1,200 rpm using a Gyro 416 G (Gyrozen, Daejeon, Korea) for 6 min and the supernatant was discarded. The cell pellets were resuspended in binding buffer consisting of 0.01 M HEPES/NaOH (pH 7.4) (Sigma-Aldrich Co.) containing 0.14 M NaCl (Sigma-Aldrich Co.), 2.5 mM CaCl_2_ (Sigma-Aldrich Co.), 5 μl propidium iodide (Sigma-Aldrich Co.), and 80 μl/ml ribonuclease A (Sigma-Aldrich Co.). After 20–30 min of incubation at room temperature in the dark, the DNA content of the cells was examined using a BD Model FACScan flow cytometer (Becton-Dickinson, San Jose, CA, USA).

### Protein extraction and western blot analysis

Cells were seeded in 100-mm culture dishes (1.0×10^6^ cells/well), incubated for 24 h, and then treated in quadruplicate with DMSO, FK-3000 (MDA-MB-231 cells, 0.5, 2.5, or 5.0 μg/ml; MCF-7 cells, 0.7, 3.5 or 7.0 μg/ml), 5.0 μM SB 239063 (both cell lines), or combination treatment (MDA-MB-231 cells, 0.5 μg/ml FK-3000 + 5.0 μM SB 239063; MCF-7 cells, 0.7 μg/ml FK-3000 + 5.0 μM SB 239063). After incubation for 45 min to 48 h, cells were harvested by trypsinization and washed twice with cold phosphate-buffered saline (PBS, Sigma-Aldrich Co.). Total protein was prepared with Pro-Prep™ (iNtRON Biotechnology, Seongnam, Korea), and the protein content of each sample was determined using the Bio-Rad DC protein assay kit (Bio-Rad, Hercules, CA, USA). Equal amounts of protein were separated by 10% SDS-polyacrylamide gel electrophoresis and transferred to a nitrocellulose membrane in Trans-Blot^®^ Transfer Medium (Bio-Rad). Membranes were incubated with anti-phospho-p38 MAPK monoclonal antibody (Cell Signaling Technology, Danvers, MA, USA; cat no. 9215), anti-phospho-CDC25C antibody (Cell Signaling Technology, cat no. 9527), anti-phospho-CDC25B antibody (Abgent, San Diego, CA, USA; AP3053a), anti-cyclin B antibody (Santa Cruz Biotechnology, Santa Cruz, CA, USA; SC-245), anti-phospho-CDC-2 antibody (Cell Signaling Technology, cat no. 9112), anti-cyclin A antibody (Santa Cruz Biotechnology, SC-751), anti-phospho-retinoblastoma (RB) antibody (Cell Signaling Technology, cat no. 9308), and anti-β-actin monoclonal antibody (Sigma-Aldrich Co., cat no. A-5316). Horseradish peroxidase-conjugated goat anti-rabbit IgG (Cayman, Ann Arbor, MI, USA; cat no. 10004301) was used as the secondary antibody. Stained bands were analyzed using the ECL detection kit (Amersham Biosciences, Buckinghamshire, UK).

### p38 MAPK phosphorylation assay

Attached cells were treated with DMSO alone, FK-3000 (MDA-MB-231 cells, 0.5 μg/ml; MCF-7 cells, 0.7 μg/ml), 5.0 μM SB 239063 (both cell lines), or combination treatment (MDA-MB-231 cells, 0.5 μg/ml FK-3000 + 5.0 μM SB 239063; MCF-7 cells, 0.7 μg/ ml FK-3000 + 5.0 μM SB 239063), and incubated for 2 h in a confocal dish (SPL Life Science, Pochoen, Korea). Cells were washed three times in cold PBS, fixed with 4% paraformaldehyde (Sigma-Aldrich Co.) at room temperature, treated with 0.5% Triton X-100, blocked with Animal-Free Blocker™ (Vector, Burlingame, CA, USA) for 1 h and incubated overnight at 4°C with anti-phospho-p38 MAPK monoclonal antibody. Cells were then incubated for 1 h with fluorescein isothio-cyanate (FITC)-conjugated goat anti-rabbit IgG (Cayman) followed by 7 μg/ml bisbenzimide H 33342 trihydrochloride (Sigma-Aldrich Co.) for nuclear staining, and photographed using an LSM510 Meta Fluorescent Microscope with Plan-Apochromat 100x/1.4 Oil DIC (Carl-Zeiss, Jena, Germany).

### Analysis of apoptosis

Attached cells were treated for 48 h with DMSO alone, FK-3000 (MDA-MB-231 cells, 0.5 μg/ml; MCF-7 cells, 0.3 μg/ml), 5.0 μM SB 239063 (both cell lines), or combination treatment (MDA-MB-231 cells, 0.5 μg/ml FK-3000 + 5.0 μM SB 239063; MCF-7 cells, 0.3 μg/ml FK-3000 + 5.0 μM SB 239063). Cells were harvested by trypsinization, washed in cold PBS, and resuspended in binding buffer consisting of 0.01 M HEPES/NaOH (pH 7.4) containing 0.14 M NaCl and 2.5 mM CaCl_2_. FITC-conjugated Annexin V (BioVision, Milpitas, CA, USA) and propidium iodide (5 μl each) (Becton-Dickinson) were added to the cells, which were gently mixed and incubated for 15 min at room temperature in the dark. Binding buffer was then added, and the cells were analyzed with BD Model FACScan (Becton-Dickinson).

### Statistical analysis

Results are expressed as mean ± standard deviation (SD). Groups were compared using Tukey’s studentized range (HSD) test with SPSS Statics (IBM, Armonk, NY, USA); p<0.01 was considered statistically significant.

## Results

### FK-3000 isolated from S. delavayi Diels inhibits proliferation of human carcinoma cell-lines MDA-MB-231 and MCF-7

**Figure 1:**
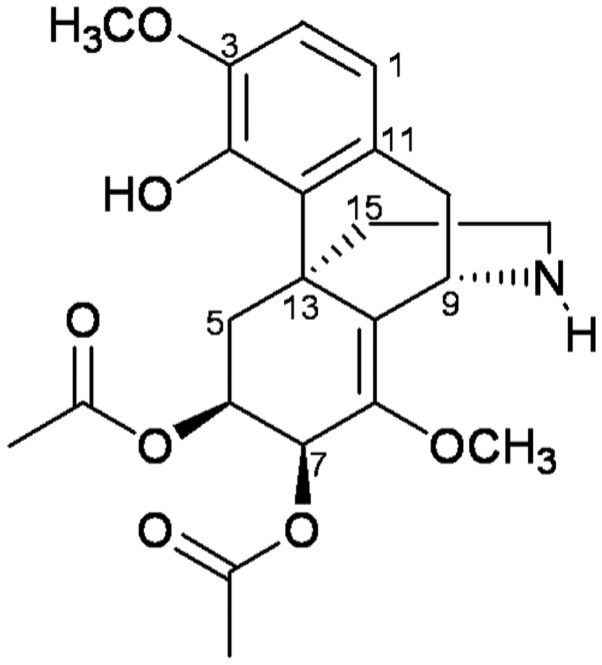
The chemical structure of 6,7-di-O-acetylsinococuline (FK-3000; molecular weight 417.4523).

We screened 509 natural products for anticancer activity and identified 14 candidates. The compound 6,7-di-O-acetylsinococuline (FK-3000) was isolated from *S. delavayi* Diels. (Fig. 1; molecular weight, 417.45), and its chemical structure was confirmed by ^1^H, ^13^C, and ^2^D NMR. The chemical structure of FK-3000 isolated from *S. delavayi* Diels. was in good agreement with the compound previously isolated from *S. cepharantha* (22).

Antiproliferative effects of FK-3000 against cancer cells have not previously been reported, we found that FK-3000 inhibited cell proliferation in a dose-and time-dependent manner in two human breast cancer cell lines. The antiproliferative effect of FK-3000 against MDA-MB-231 cells (24 h IC_50_, 0.89 μg/ml; 48 h IC_50_, 0.52 μg/ml) was greater than its effect against MCF-7 cells (24 h IC_50_, 2.53 μg/ml; 48 h IC_50_, 0.77 μg/ml).

### FK-3000 arrests MDA-MB-231 and MCF-7 cells at G_2_/M phase

**Figure 2:**
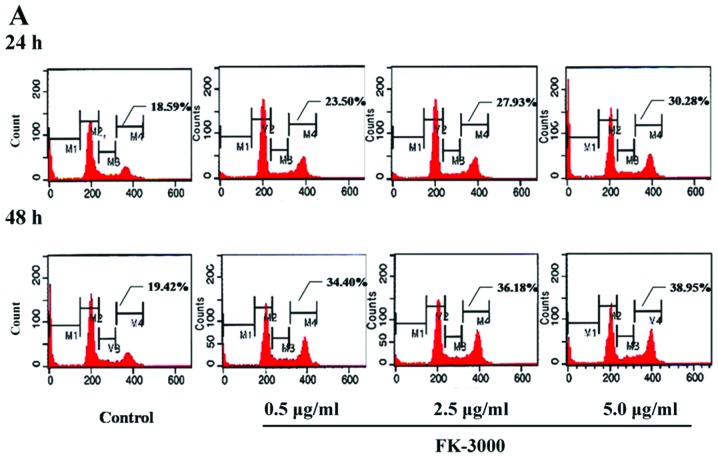

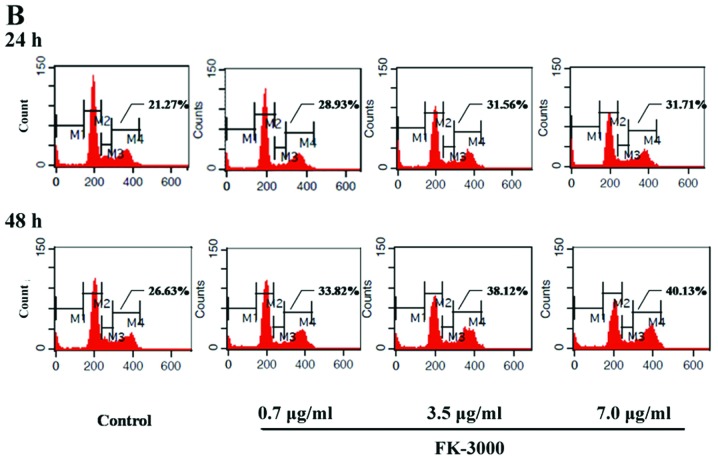
FK-3000 induces G_2_/M phase arrest in the human breast cancer cell lines MDA-MB-231 and MCF-7 in a dose- and time-dependent manner. (A) The 48 h IC_50_ of FK-3000 for MDA-MB-231 was 0.52 μg/ml. Cell cycle distribution was analyzed at 24 and 48 h after treatment with DMSO (vehicle control) or FK-3000 (0.5, 2.5, or 5.0 μg/ml). FK-3000 increased the percentage of cells in G_2_/M phase arrest from 18.59 to 30.28% at 24 h and from 19.42 to 38.95% at 48 h. (B) The 48 h IC_50_ of FK-3000 for MCF-7 cells was 0.77 μg/ml. Cell distribution was analyzed at 24 and 48 h after treatment with DMSO (vehicle control) or FK-3000 (0.7, 3.5, or 7.0 μg/ml). FK-3000 increased the percentage of cells in G_2_/M phase arrest from 21.27 to 31.71% at 24 h and from 26.63 to 40.13% at 48 h.

Carcinogenesis is caused by cell cycle deregulation, typically an increase in positive regulators such as CDKs and/or decrease in negative regulators such as cyclin D1, CDK4, cyclin E, cyclin A, and Wee1. Because the cell cycle is no longer controlled, cell proliferation is excessive (6). We therefore measured the effect of FK-3000 on cell cycle regulation in MDA-MB-231 and MCF-7 cells. Doses were based on the 48 h IC_50_ of FK-3000 for each cell line, corresponding to 1× IC_50_ to 10× IC_50_ for each cell line (MDA-MB-231, 0.5–5.0 μg/ml; MCF-7, 0.7–7.0 μg/ml). As shown in Fig. 2A and B, FK-3000 treatment resulted in G_2_/M phase arrest in a time- and dose-dependent manner. In MDA-MB-231 cells treated with 1× IC_50_ FK-3000 for 24 h, the percentage of G_2_/M phase arrested cells was 23.50%, increasing to 38.95% after 48-h treatment with 10× IC_50_ FK-3000. In MCF-7 cells treated with 1× IC_50_ FK-3000 for 24 h, the percentage of G_2_/M phase arrested cells was 28.93%, increasing to 40.13% after 48-h treatment with 10× IC_50_ FK-3000.

### FK-3000 induces dephosphorylation of CDC25 through p38 MAPK signaling

In cancer cells, levels of phosphorylated p38 MAPK proteins are low whereas phosphorylated CDC25B protein levels are high. CDC25B plays a key role in G_2_/M phase transition and CDC2 activation (23); phosphorylation of CDC25B is an important step leading to proliferation and metastasis of neoplastic cells. P38 MAPK induces G_2_/M arrest by inhibiting CDC25B phosphorylation and blocking participation of the CDC2/cyclin B complex in G_2_/M transition (20,21).

**Figure 3:**
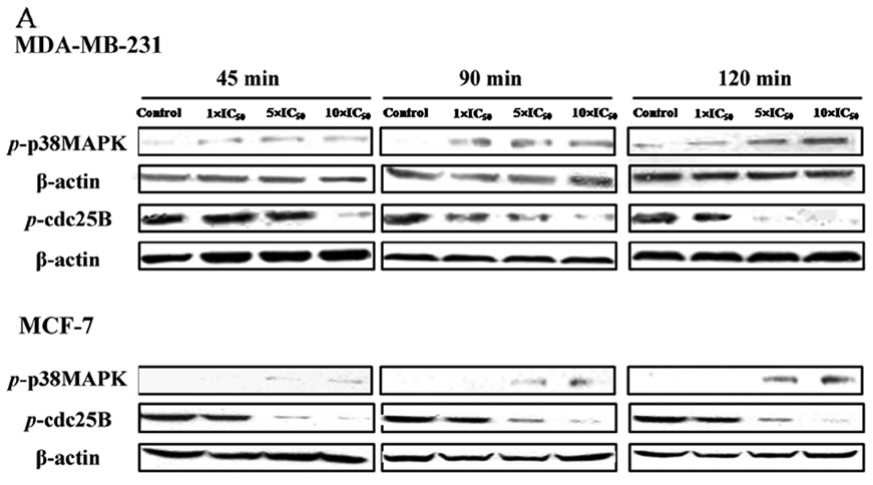

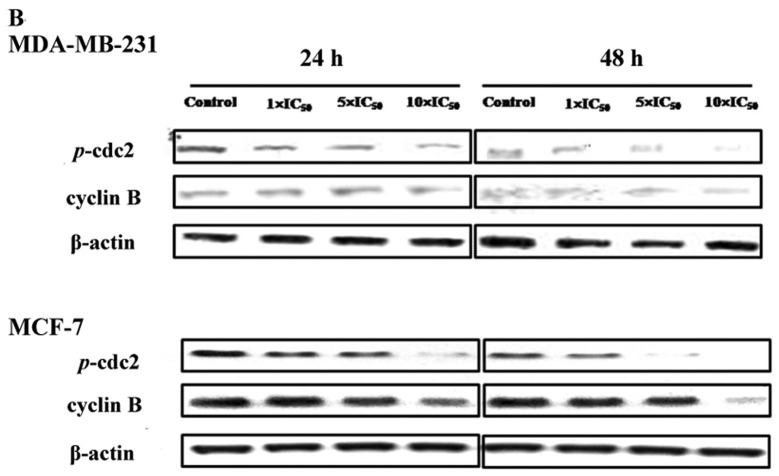
FK-3000 decreased levels of phospho-CDC-2 and cyclin B through p38 MAPK phosphorylation and CDC25B dephosphorylation. (A) FK-3000 upregulated p38 MAPK phosphorylation and downregulated CDC25B phosphorylation in both MDA-MB-231 and MCF-7 human breast cancer cells in a dose-and time-dependent manner. (B) FK-3000 decreased the phosphorylation of CDC2 in a dose- and time-dependent manner. A 48-h treatment with FK-3000 (MDA-MB-231 cells, 10× IC_50_ FK-3000; MCF-7 cells, 5× IC_50_ to 10× IC_50_ FK-3000) completely abolished CDC2 phosphorylation. Although 24-h treatment with FK-3000 slightly increased cyclin B levels in MD-MB-231 cells, at 48 h cyclin B protein levels decreased in a dose-dependent manner. In MCF-7 cells, cyclin B was decreased only by treatment with 10× IC_50_ FK-3000.

As shown in Fig. 3A, FK-3000 increased phosphorylation of p38 MAPK and decreased phosphorylation of CDC25B in both MDA-MB-231 and MCF-7 cell lines. Levels of phosphorylated p38 MAPK increased in a dose- and time-dependent manner in MCF-7 cells, whereas the level of phosphorylated p38 MAPK at 90 min differed from that of other time points in MDA-MB-231 cells. In MDA-MB-231 cells, phosphorylation of CDC25B in cells treated with 1× IC_50_ FK-3000 was similar to that of the 5× IC_50_ group at 45 min, but was almost completely abolished by 90-min treatment with 10× IC_50_ FK-3000 and 120-min treatment with 5× IC_50_ FK-3000. In MCF-7 cells, FK-3000 significantly reduced phospho-CDC25B in a dose-and time-dependent manner, and 10× IC_50_ FK-3000 almost completely suppressed phosphorylation of CDC25B at all time points. These results suggest that FK-3000 inhibits CDC25B through p38 MAPK activation.

We next determined the effect of FK-3000 on the G_2_/M phase regulatory factors and related proteins CDC-2, cyclin A, cyclin B, and RB. With the exception of cyclin B and phospho-CDC-2, we did not observe changes in these proteins (data not shown). Cyclin B levels were not altered by 24 h FK-3000 treatment in either MDA-MB-231 or MCF-7 cells, except in cells treated with 10× IC_50_ FK-3000; however, this increase was attenuated at 48 h, and cyclin B was barely detectable after 48-h treatment with 10× IC_50_ FK-3000 in both cell lines (Fig. 3B). FK-3000 decreased phosphorylation of CDC2 in a dose- and time-dependent manner, and 48-h treatment with 10× IC_50_ FK-3000 in MDA-MB-231 cells and 5× IC_50_ or 10× IC_50_ FK-3000 in MCF-7 cell completely abolished phosphorylation of CDC2 (Fig. 3B).

### p38 MAPK inhibition attenuated the antiproliferative action of FK-3000 but did not completely block FK-3000-induced apoptosis

CDC25B phosphorylation is regulated by p38 MAPK, which also blocks participation of the CDC2/cyclin B complex in G_2_/M transition (20,21). Phosphorylation of p38 MAPK plays a role in cell death, cell differentiation, and cell cycle progression. Following DNA damage, phospho-p38 MAPK translocates from the cytoplasm into the nucleus (24), where accumulation of phospho-p38 MAPK triggers G_2_/M phase arrest and DNA repair.

**Figure 4:**
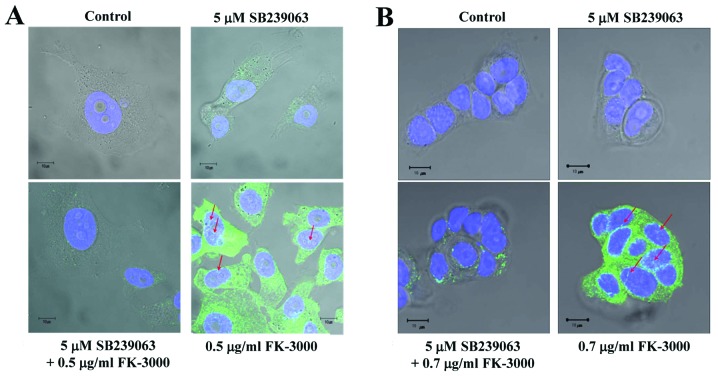

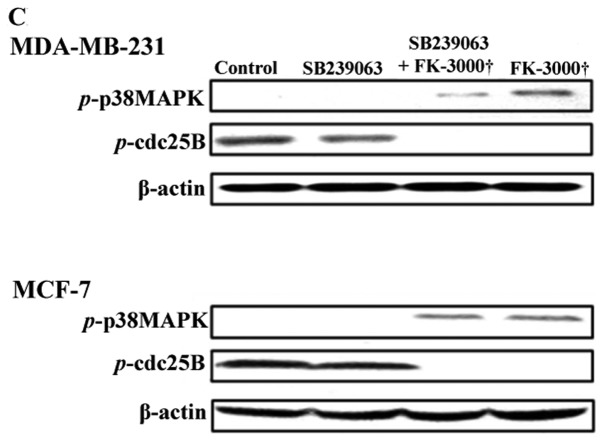

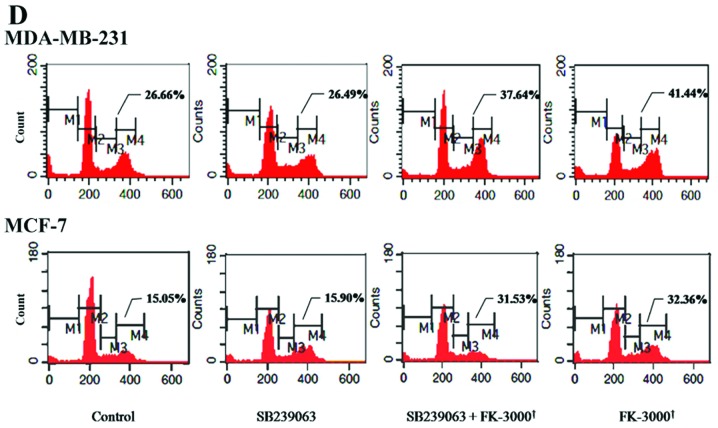

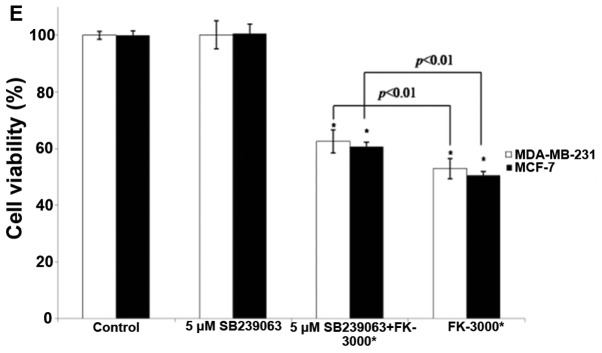

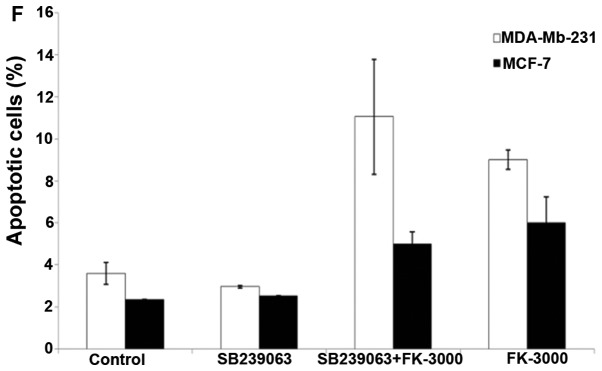
FK-3000 inhibits cell proliferation by inducing p38 MAPK phosphorylation. (A and B) Phospho-p38 MAPK levels were significantly higher in the cytoplasm and the nucleus after 90-min treatment with FK-3000 in both MDA-MB-231 (A) and MCF-7 cells (B); however, p38 MAPK phosphorylation and nuclear translocation were almost completely suppressed by the p38 MAPK inhibitor SB 239063 (red arrows indicate the phosphorylated form of p38 MAPK in the nucleus). (C) At 90 min, SB 239063 reduced FK-3000-stimulated p38 MAPK phosphorylation, but could not suppress FK-3000-mediated CDC25B dephosphorylation. (D) At 24 h, SB 239063 reduced but could not completely reverse FK-3000-induced G_2_/M phase arrest. (E) At 48 h, SB 239063 attenuated the antiproliferative effect of FK-3000. (F) At 48 h, the percentage of apoptotic cells was significantly higher in FK-3000 treated cells (SB 239063 + FK-3000 or FK-3000 only) compared with cells not exposed to FK-3000 (vehicle control or SB 239063 only; ^*^p<0.01 (Tukey’s studentized range test)]. ^†^FK-3000 treatment (0.5 μg/ml) for MDA-MB-231 cells and FK-3000 treatment (0.7 μg/ml) for MCF-7 cells.

We assumed that FK-3000 induced p38 MAPK phosphorylation and then suppressed CDC25B phosphorylation. Our results showed that a 90-min FK-3000 treatment stimulated p38 MAPK phosphorylation and nuclear translocation in MDA-MB-231 and MCF-7 cells (Fig. 4A and B), and this effect was suppressed by SB 239063, a potent and selective inhibitor of p38 MAPK (25). We compared phospho-p38 MAPK and phospho-CDC25B levels in FK-3000 treated cells with that of untreated cells at 90 min (Fig. 4C). Phosphorylation of CDC25B was abolished in cells treated with FK-3000 in the presence or absence of SB 239063. Together, these findings indicate that FK-3000 inhibits CDC25B phosphorylation directly as well as indirectly through p38 MAPK phosphorylation.

To evaluate the mechanism of cell cycle arrest by FK-3000, we analyzed the cell cycle distribution of treated cells. Although the distribution of cells treated with SB 239063 was similar to that of the vehicle control, SB 239063 could not completely reverse FK-3000-induced G_2_/M phase arrest (Fig. 4D).

To confirm that FK-3000 inhibited cell proliferation through p38 MAPK activation, we evaluated whether the p38 MAPK inhibitor SB 239063 could rescue the antiproliferative effect of FK-3000. Our results showed that SB 239063 attenuated but could not completely block the antiproliferative action of FK-3000. SB 239063 increased viability from 52.93 to 62.52% in FK-3000-treated MDA-MB-231 cells and increased viability from 50.59 to 60.63% in FK-3000-treated MCF-7 cells (Fig. 4E). As shown in Fig. 4E, the viability of cells treated with both SB 239063 and FK-3000 (77.69% in MDA-MB-231, 60.63% in MCF-7) did not fully recover to the level of control cells, suggesting that FK-3000 inhibits cell proliferation by an additional mechanism besides G_2_/M phase arrest through p38 MAPK phosphorylation and CDC25B dephosphorylation. We therefore analyzed the effect of SB 239063 on the rate of apoptosis in cells treated with FK-3000 (Fig. 4F). Apoptosis in cells treated with FK-3000 (SB 239063 + FK-3000 cotreatment or FK-3000 only) was significantly higher than that of cells treated with the vehicle control or SB 239063 only. Thus, FK-3000 appears to induce apoptosis by a pathway independent of the p38 MAPK-CDC25B pathway.

Taken together, these findings indicate that FK-3000 is a promising anticancer drug candidate that exerts its antiproliferative activity through two pathways: induction of G_2_/M phase arrest by p38 MAPK-CDC25B-CDC2-cyclin B modulation and stimulation of apoptosis independent of the p38 MAPK-CDC25B pathway.

## Discussion

Cell cycle regulatory factors and related proteins (e.g., cyclin A, cyclin B, CDC2, CDC25A, CDC25B, CDC25C and p38 MAPK) are associated with G_2_/M transition; in particular, the CDC2-cyclin B heterodimeric complex regulates entry into mitosis (26). We found that FK-3000 induced G_2_/M phase arrest in the human breast carcinoma cell lines MDA-MB-231 and MCF-7 in a dose- and time-dependent manner. Further, phospho-CDC2 levels were significantly decreased after 24 h and cyclin B levels were decreased after 48 h, and phospho-p38 MAPK was upregulated, whereas phospho-CDC25B was downregulated in a dose- and time-dependent manner. Taken together, our findings suggest that FK-3000 induces G_2_/M arrest by inhibiting CDC2 activation via p38 MAPK phosphorylation and CDC25B dephosphorylation. To confirm these results, we evaluated the ability of the selective p38 MAPK inhibitor SB 239063 to block the antiproliferative action of FK-3000. SB 239063 increased viability from 52.93 to 62.52% in FK-3000-treated MDA-MB-231 cells and from 50.59 to 60.63% in FK-3000-treated MCF-7 cells. Moreover, SB 239063 inhibited FK-3000-induced p38 MAPK phosphorylation and nuclear accumulation (Fig. 4A–C).

However, SB 239063 did not completely rescue the effects of FK-3000, suggesting the involvement of another pathway in the antiproliferative action of FK-3000. Although SB 239063 suppressed FK-3000-induced p38 MAPK phosphorylation, it did not inhibit apoptosis (Fig. 4F). We therefore propose that FK-3000 exerts its cytostatic effect through p38 MAPK activation and its cytotoxic effect through apoptosis. CDC25B has been proposed as a target for the development of anticancer agents (14,23). EK-6136 is a synthetic CDC25B inhibitor that inhibits cell proliferation in MCF-7 (48 h IC_50_, 7.2±1.0 μM), HT-29 (48 h IC_50_, 8.4±1.0 μM), and A549 cells (48 h IC_50_, 7.7±1.0 μM) (27). BN82002 is a synthetic pan-CDC25 inhibitor that reduces proliferation of the carcinoma cell lines Mia PaCa-2, DU-145, U-87 MG, LNCaP, HT-29, and U2OS, with 96-h IC_50_ values in the range 7.2–32.6 μM (15). Another synthetic pan-CDC25 inhibitor, naphthofurandione 3-benzoyl-naphtho[1,2-*b*]furan-4,5-dione, inhibits cell proliferation in PC-3 cells (96 h IC_50_, 6.5 μM) and MDA-MB-435 cells (96 h IC_50_, 1.2 μM) (17). FK-3000 suppresses activation of CDC25B but not CDC25C. Compared with the previously described CDC25 inhibitors, FK-3000 is a more potent inhibitor of proliferation in various cell lines and appears to be safe as assessed by animal studies at doses <10 mg/kg of body weight, administered intraperitoneally once a day for 5 days (data not shown).

Cell cycle regulators may be positive (e.g., CDKs, cyclins) or negative [e.g., INK4 family (p16^ink4a^, p15^ink4b^, p18^ink4c^, and p19^ink4d^), p21^waf1^, p27^Kip1^, and p57^Kip2^] (28–30). Carcinogenesis is the result of an imbalance between these positive and negative regulatory factors; therefore, modulating these proteins is a common therapeutic strategy against neoplasms. Recent studies have evaluated CDK modulators as anticancer agents. For example, the staurosporine analogue, 7-hydroxystaurosporine (UCN-01), is in phase I/II clinical trials for leukemia, lymphoma, ovarian epithelial, primary peritoneal or fallopian tube cancer, and unspecified solid tumors (31), and the flavonoid flavopiridol is in phase I/II clinical trials for non-Hodgkin lymphoma, renal, prostate, colon, and gastric cancers (32,33). UCN-01 induces CDC2 dephosphorylation at Tyr-15, promoting early entry into mitosis and ultimately inducing arrest at the G_2_/M phase (32). The 24-h IC_50_ of UCN-01 in MDA-MB-231 cells is ~1 μM (34). Like UCN-01, FK-3000 dephosphorylates CDC2 at Tyr-15, activates CDC/cyclin B, and facilitates initiation of mitosis. Although flow cytometric analysis in the present study showed that FK-3000 induced G_2_/M phase arrest in MDA-MB-231 and MCF-7 cells, most of these cells may be in mitosis.

**Figure 5:**
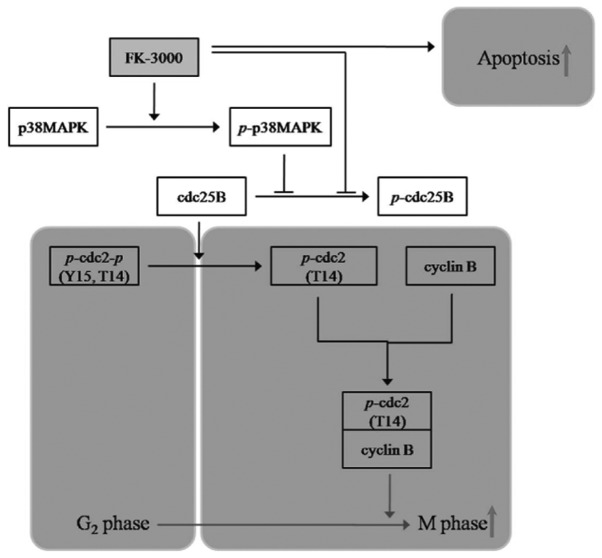
Schematic showing the antiproliferative pathways of FK-3000. FK-3000 stimulates p38 MAPK phosphorylation, and activated p38 MAPK downregulates phosphorylation of CDC25B both directly and indirectly. The decrease in phospho-CDC25B increases the relative amount of unphosphorylated CDC25B, which dephosphorylates one (Y15) of the two phosphorylated tyrosine residues of phosphorylated CDC2 (Y15 and T14). Phospho-CDC2 (pT14) then forms a heterodimeric complex with cyclin B, leading to an increase in the number of cells in M phase. FK-3000 also induces apoptosis through a p38 MAPK-independent pathway. Thus, FK-3000 promotes at least two antiproliferative actions in MDA-MB-231 and MCF-7 cells: G_2_/M phase arrest (in particular M phase arrest) and apoptosis.

We demonstrated that FK-3000 exerts an antiproliferative effect through two pathways: i) G_2_/M phase arrest via downregulation of cyclin B and phospho-CDC2 by dephosphorylation of CDC25B and phosphorylation of p38 MAPK; and ii) p38 MAPK-independent induction of apoptosis (Fig. 5). Although further studies are needed to evaluate FK-3000 in other cancer cell types and elucidate the antiproliferative mechanisms, therapeutic index, and margin of safety, our findings indicate that FK-3000 is a promising anticancer agent.
